# Promoter methylation patterns of *ABCB1*, *ABCC1* and *ABCG2* in human cancer cell lines, multidrug-resistant cell models and tumor, tumor-adjacent and tumor-distant tissues from breast cancer patients

**DOI:** 10.18632/oncotarget.12332

**Published:** 2016-09-28

**Authors:** Melanie Spitzwieser, Christine Pirker, Bettina Koblmüller, Georg Pfeiler, Stefan Hacker, Walter Berger, Petra Heffeter, Margit Cichna-Markl

**Affiliations:** ^1^ Department of Analytical Chemistry, University of Vienna, Vienna, Austria; ^2^ Department of Medicine I, Institute of Cancer Research and Comprehensive Cancer Center of the Medical University, Medical University of Vienna, Vienna, Austria; ^3^ Department of Obstetrics and Gynecology, Division of Gynecology and Gynecological Oncology, Medical University of Vienna, Vienna, Austria; ^4^ Department of Plastic and Reconstructive Surgery, Medical University of Vienna, Vienna, Austria

**Keywords:** ABC transporter, multidrug resistance, DNA methylation, bisulfite pyrosequencing, breast cancer

## Abstract

Overexpression of ABCB1, ABCC1 and ABCG2 in tumor tissues is considered a major cause of limited efficacy of anticancer drugs. Gene expression of ABC transporters is regulated by multiple mechanisms, including changes in the DNA methylation status. Most of the studies published so far only report promoter methylation levels for either *ABCB1* or *ABCG2*, and data on the methylation status for *ABCC1* are scarce. Thus, we determined the promoter methylation patterns of *ABCB1, ABCC1* and *ABCG2* in 19 human cancer cell lines. In order to contribute to the elucidation of the role of DNA methylation changes in acquisition of a multidrug resistant (MDR) phenotype, we also analyzed the promoter methylation patterns in drug-resistant sublines of the cancer cell lines GLC-4, SW1573, KB-3-1 and HL-60. In addition, we investigated if aberrant promoter methylation levels of *ABCB1, ABCC1* and *ABCG2* occur in tumor and tumor-surrounding tissues from breast cancer patients.

Our data indicates that hypomethylation of the *ABCC1* promoter is not cancer type-specific but occurs in cancer cell lines of different origins. Promoter methylation was found to be an important mechanism in gene regulation of *ABCB1* in parental cancer cell lines and their drug-resistant sublines. Overexpression of ABCC1 in MDR cell models turned out to be mediated by gene amplification, not by changes in the promoter methylation status of *ABCC1*. In contrast to the promoters of *ABCC1* and *ABCG2*, the promoter of *ABCB1* was significantly higher methylated in tumor tissues than in tumor-adjacent and tumor-distant tissues from breast cancer patients.

## INTRODUCTION

Chemotherapy, surgery and radiation therapy are today's main pillars of cancer treatment. The efficacy of chemotherapy is, however, limited due to drug resistance. For some types of cancer, e.g. cancers derived from colon, kidney, adrenal gland, liver and pancreas, resistance often exists even before chemotherapy started (intrinsic resistance). Other types of cancer, e.g. breast and small cell lung cancer, frequently develop resistance after an initial positive response (acquired resistance) [1]. Multidrug resistance (MDR), the simultaneous resistance to a broad range of structurally unrelated drugs with different modes of action, is a particular challenge in cancer treatment [2].

Chemotherapy resistance can be caused by mechanisms in the cancer cells and/or by characteristics of the tumor microenvironment [3]. Microenvironment-related factors include the composition of the extracellular matrix, cell-cell interactions and the vasculature [4, 5]. Intracellularly, processes that limit drug uptake or increase drug inactivation, drug efflux or the repair of DNA lesions caused by cancer treatment reduce the efficacy of chemotherapy [6].

By functioning as efflux pumps, ATP-binding cassette (ABC) transporters play a crucial role in MDR of cancers. Based on their sequence homology and the number and location of transmembrane domains, the members of the human ABC transporter superfamily have been grouped into seven subfamilies (ABCA - ABCG) [7]. ABCB1, ABCC1 and ABCG2 are the ABC transporters most frequently been associated with MDR in cancers [8, 9].

In healthy tissues, ABCB1, ABCC1 and ABCG2 are physiologically relevant because they protect the body from a variety of toxic endogenous and exogenous compounds by transporting them across cell membranes, even against very steep concentration gradients [10–12]. ABCB1 can transport neutral and cationic hydrophobic compounds [13, 14], whereas ABCC1 predominantly transports xenobiotic organic anions conjugated with glutathione, glucuronic acid or sulphate [15]. In addition to conjugated organic anions, ABCG2 has been reported to transport phosphorylated nucleosides and nucleotides [16].

Overexpression of ABCB1, ABCC1 and/or ABCG2 in tumor tissues is considered a major cause of limited efficacy of anticancer drugs. Increased levels of ABCB1 are commonly found in intrinsically resistant cancers [17]. Overexpression of ABCC1 has been linked to MDR in small cell lung carcinoma, prostate and breast cancer as well as childhood neuroblastoma [18]. High levels of ABCG2 have been associated with MDR in acute lymphoblastic leukemia, chronic myeloid leukemia and non-small cell lung cancer. Increasing evidence suggests that ABCG2 is involved in MDR of cancer stem cells [19, 20].

Although the amino acid sequences of ABCB1, ABCC1 and ABCG2 are quite different, their resistance profiles are significantly overlapping [21, 22]. Each of the three ABC transporters confers resistance to anthracyclines (e.g. doxorubicin and daunorubicin), epipodophyllotoxins (e.g. etoposide and teniposide) and campothecins. Cancer cells overexpressing ABCB1 and/or ABCC1 are also resistant to *Vinca* alkaloids (e.g. vincristine) and colchicine. Among the three ABC transporters, ABCB1 is the only one conferring resistance to taxanes (e.g. paclitaxel and docetaxel) [9].

Gene expression of *ABCB1*, *ABCC1* and *ABCG2* is regulated by multiple mechanisms at both the transcriptional and post-transcriptional level [23–25]. In addition, epigenetic mechanisms including changes in the DNA methylation status and histone modifications are known to play a role by regulating the structure of chromatin. Hypomethylation of the *ABCB1* promoter has been detected e.g. in MDR sublines of the human T-cell leukemia cell line CCRF-CEM [26] and the breast cancer cell line MCF-7 [27, 28], obtained by selecting the parental cells for resistance to doxorubicin. Hypomethylation of the *ABCG2* promoter has for example been found in ABCG2-overexpressing sublines of MCF-7, CCRF-CEM, IGROV1 (ovarian carcinoma) and A549 (non-small cell lung cancer) cells [29]. To our knowledge, only one study has investigated the promoter methylation status of *ABCC1* [30]. Methylation levels of *ABCB1, ABCC1* and *ABCG2* have been determined in the pancreatic cancer cell line SW1990 and its drug-resistant subline SW1990/GZ, obtained by selecting SW1990 for resistance to gemcitabine. Although expression of the three ABC transporters was significantly higher in SW1990/GZ than in SW1990 cells, the promoters of *ABCB1*, *ABCC1* and *ABCG2* were found to be hypomethylated, in both, the MDR subline and the parental cell line [30]. With the exception of Chen *et al.* [30] and Oberstadt *et al.* who investigated the methylation status of *ABCB1* and *ABCG2* in glioblastoma [31], studies reporting DNA methylation levels in cancer cell lines and/or clinical tumor samples focused on either *ABCB1* [27, 28, 32–40] or *ABCG2* [29, 41–43].

In the present study we aimed to enlarge the database by determining the promoter methylation patterns of *ABCB1*, *ABCC1* and *ABCG2* in cancer cell lines derived from different types of cancer, MDR cell models as well as tumor, tumor-adjacent and tumor-distant tissues from breast cancer patients. First of all, we were interested if *ABCC1* hypomethylation is cancer type-specific or also occurs in other types of cancer than pancreatic cancer. Knowledge of the promoter methylation patterns of *ABCB1*, *ABCC1* and *ABCG2* in cancer cell lines will be helpful, e.g. in selecting an appropriate cell line for investigating the mode of action and/or testing the efficacy of potential chemotherapeutic drugs. In MDR sublines of the small cell lung cancer cell line GLC-4, the non-small cell lung cancer cell line SW1573, the epidermal cervical cancer cell line KB-3-1 and the promyelocytic leukemia cell line HL-60, the promoter methylation patterns of the three ABC transporters were determined in order to contribute to elucidation of the role of DNA methylation changes in acquisition of a MDR phenotype. Data obtained by array comparative genomic hybridization (array CGH), whole genome gene expression arrays and Western Blots was used to investigate if the promoter methylation status is linked to copy number variation and expression at the gene and/or protein level.

A few papers indicate that hypermethylation of the *ABCB1* promoter is a frequent event in breast cancer [34, 36, 38], so far, data on promoter methylation of *ABCC1* and *ABCG2* has, however, not been published. Changes in the DNA methylation status are known to be an early event in carcinogenesis. They have been detected not only in tumors but also in tumor-adjacent tissue that appeared histologically normal. The presence of molecular abnormalities in tumor-surrounding tissues is called field cancerization or field defect [44]. We determined the methylation patterns in tumor, tumor-adjacent and tumor-distant tissues from the same breast cancer patients in order to investigate if aberrant promoter methylation levels of *ABCB1*, *ABCC1* and *ABCG2* can be used as indicator for detection of field cancerization in breast cancer. We also evaluated if there is a correlation between the promoter methylation status of *ABCB1*, *ABCC1* and *ABCG2* and if the methylation levels are associated with any of the clinicopathological parameters.

## RESULTS

### Development and validation of bisulfite pyrosequencing methods for *ABCB1*, *ABCC1* and *ABCG2*

**Figure 1:**
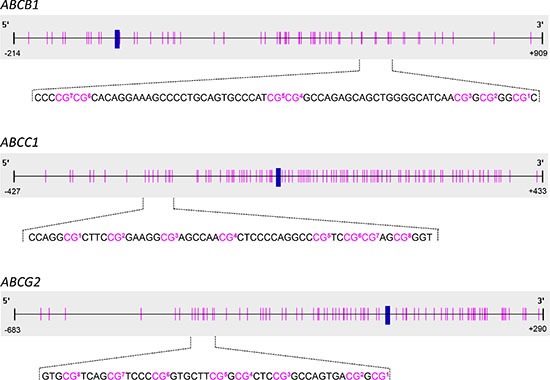
Schematic representation of the positions of the CpGs targeted by bisulfite pyrosequencing analysis The transcription start site (TSS, +1) is indicated by a blue vertical bar, the positions of the CpGs by pink vertical lines. In the sequences to analyze, the CpGs are numbered according to their order in the respective pyrogram.

**Figure 2:**
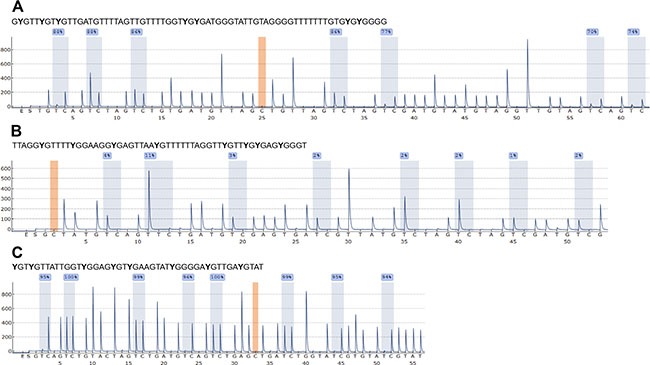
Representative pyrograms for *ABCB1* (A), *ABCC1* (B) and *ABCG2* (C) obtained with in-house developed bisulfite PSQ methods Peaks highlighted in blue indicate the methylation status of the CpGs in the sequence to analyze. The position highlighted in orange serves as control for complete bisulfite conversion. Pyrograms were obtained by analyzing the prostate cancer cell line PC-3.

Bisulfite pyrosequencing (PSQ) methods for *ABCB1*, *ABCC1* and *ABCG2* were developed in-house. For each method, the concentrations of the forward and reverse primer and the annealing temperature were optimized. PCR conditions resulting in the highest amount of specific PCR product without leading to the formation of unspecific products are summarized in Table 1. Figure 1 shows the cytosine-phosphatidyl-guanosine (CpG) island, the transcription start site (TSS), the position of the CpGs targeted by the bisulfite PSQ method and the sequence to analyze for *ABCB1*, *ABCC1* and *ABCG2*. The *ABCB1* method allows determining the methylation status of seven CpGs downstream of the TSS (+524, +526, +554, +556, +580, +583, +587). The methods for *ABCC1* and *ABCG2* target eight CpGs upstream of the TSS (*ABCC1*: −214, −208, −201, −193, −180, −176, −174, −170; *ABCG2*: −357, −351, −345, −337, −334, −329, −319, −316). Representative pyrograms are shown in Figure 2. In the pyrogram for *ABCC1*, the variable positions (Y = C or T) appear in the same order as in the sequence to analyze shown in Figure 1, because the forward strand of the DNA was used for primer design. Since in case of *ABCB1* and *ABCG2*, the primers were designed by using the reverse strand, the sequence reaction was performed in the opposite direction, as indicated by the numbering of the CpGs in the sequences to analyze in Figure 1.

**Table 1:** Bisulfite pyrosequencing analysis

Gene	Primer sequence (5′→3′)	Chromosome	GenBank accession number	Amplicon length [bp]	No. and position of CpGs analyzed	Primer concentration [nM]	T_a_ [°C]
*ABCB1*	F: GTTGGAGGT GAGATTAATTTT	7	NG_011513.1	162	7 [117888–117952]	200	58.3
	R: [Btn]AAACCCC CAACTCTACCT						
	S: GAGAGTAGTAA GAGGGA						
*ABCC1*	F: TTTATAGGATGA AATGAGGGTATAGT	16	NG_028268.1	284	8 [4786–4831]	400	59.8
	R: [Btn]AACAACCCA ACCAACCACCTCT						
	S: GTGTGTGGTTTT AAAGATT						
*ABCG2*	F: GTTTGATTTAGTT GGGTTTGG	4	NG_032067.2	122	8 [77051–77093]	400	54.8
	R: [Btn]AACCACCC ATTTAACTTACTCT						
	S: ATTTAGTTGGGTT TGGT						

Each of the PSQ methods was found to be applicable to determine the methylation status in cancer cell lines and biopsy samples from breast cancer patients. The limit of quantification (LOQ), determined by repeatedly analyzing bisulfite-treated unmethylated control DNA, was found to be 5%.

### Promoter methylation patterns of *ABCB1*, *ABCC1* and *ABCG2* in cancer cell lines

**Figure 3:**
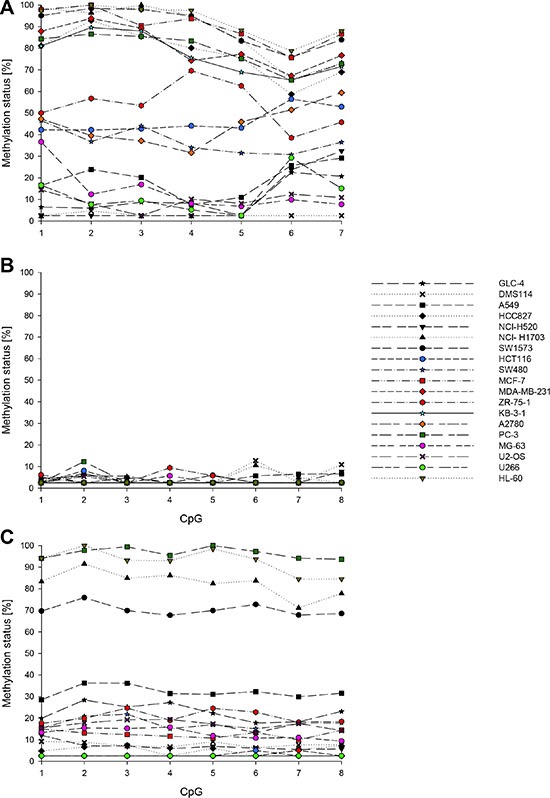
Promoter methylation patterns of *ABCB1* (A), *ABCC1* (B) and *ABCG2* (C) in 19 cancer cell lines Mean values of at least two technical replicates.

The promoter methylation status of *ABCB1*, *ABCC1* and *ABCG2* (Figure 1) was determined in 19 cancer cell lines: the small cell lung carcinoma DMS114 and GLC- 4; the non-small cell lung carcinoma A549, HCC827, NCI-H520, NCI-H1703 and SW1573; the colorectal carcinoma HCT116 and SW480; the breast cancer cell models MCF-7, MDA-MB-231 and ZR- 75– 1; the cervical cancer cell line KB-3-1; the ovarian carcinoma A2780; the prostate cancer cell line PC-3; the osteosarcoma MG-63 and U2-OS; the multiple myeloma U266 and the promyelocytic leukemia HL- 60. Overall, the cell lines showed big differences in the extent of promoter methylation of *ABCB1* (Figure 3A). According to their methylation status, the cancer cell lines could be divided into three groups. In seven cancer cell lines (GLC-4, DMS114, A549, NCI-H520, U2-OS, MG-63 and U266), the average methylation status (across the seven CpGs investigated) was found to be < 20%, in DMS114 cells it was even < LOQ (< 5%). In eight cell lines (KB-3-1, HCC827, SW1573, NCI-H1703, MDA-MB-231, MCF-7, PC-3 and HL-60), the target region was highly methylated (average methylation status > 75%). Intermediate methylation levels ranging from 37 to 54% were found for SW480, HCT116, A2780 and ZR-75-1 cells. In several cancer cell lines, e.g. ZR-75-1, A549 and A2780, the promoter region of *ABCB1* was methylated rather heterogeneously.

In none of the 19 cancer cell lines the average methylation status of the *ABCC1* promoter (across the eight CpGs investigated) was found to be ≥ 5% (Figure 3B). Moreover, the average methylation status of the *ABCG2* promoter (across the eight CpGs in the sequence to analyze) was even < LOQ in five cancer cell lines (A2780, KB-3-1, MDA-MB-231, HCT116 and U266) (Figure 3C). In four cell lines (PC-3, NCI-H1703, SW1573 and HL-60), the eight CpGs were highly methylated (methylation status ranging from 70 to 97%). In the remaining ten cell lines, the average methylation status was between 5 and 32%.

### Copy number variation of *ABCB1*, *ABCC1* and *ABCG2* in cancer cell lines

**Figure 4:**
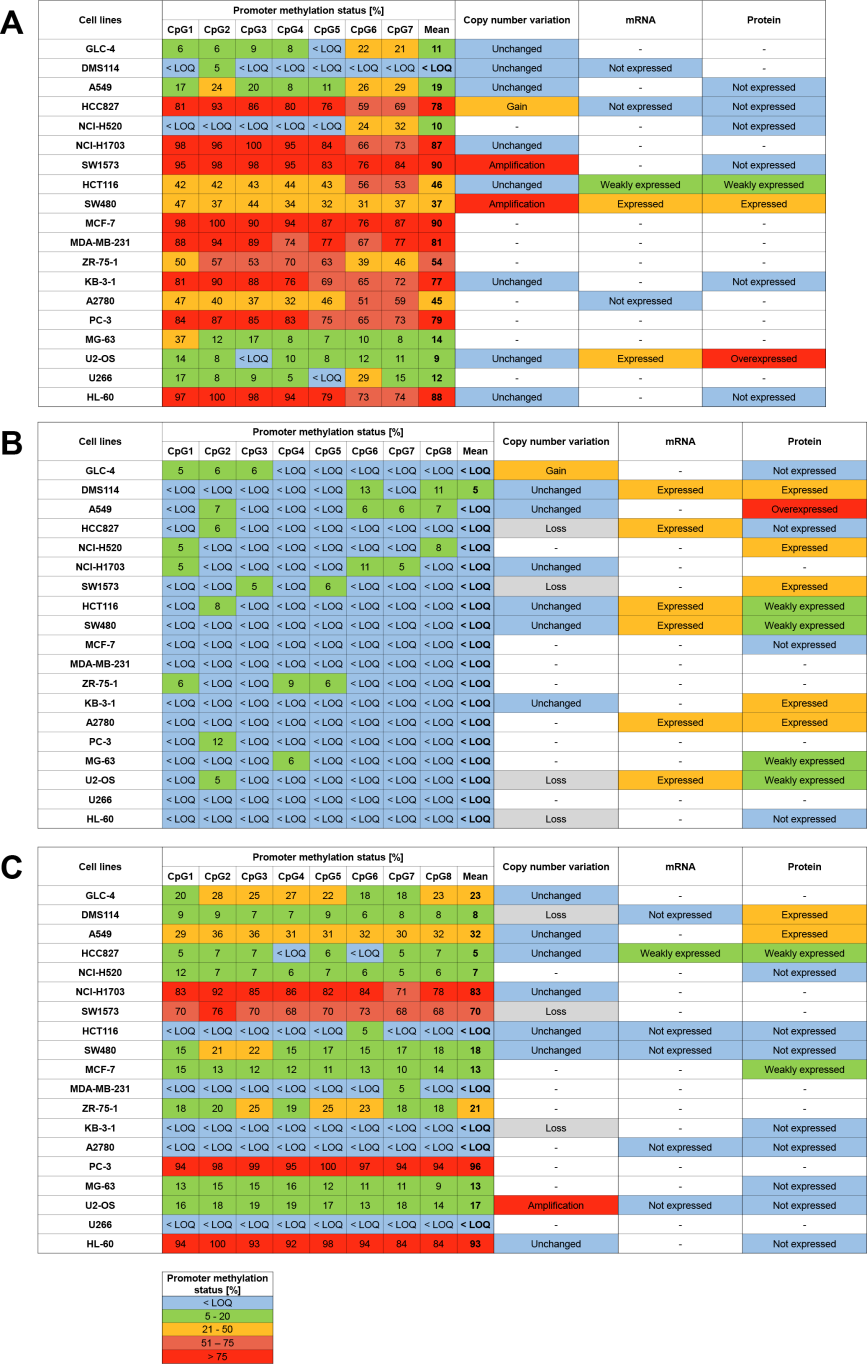
Association of promoter methylation status, copy number variation and expression of *ABCB1* (A), *ABCC1* (B) and *ABCG2* (C) in cancer cell lines Limit of quantification (LOQ): 5%. “-” not determined.

Copy number variation analyses by array CGH indicated that the *ABCB1* gene is amplified in SW1573 and SW480 cells (Figure 4A), whereas U2-OS cells show amplification of *ABCG2* (Figure 4C). An increase (gain) in the copy number of *ABCB1* and *ABCC1* was observed in HCC827 and GLC-4 cells, respectively.

### mRNA and protein expression of ABCB1, ABCC1 and ABCG2 in cancer cell lines

For some cell lines, expression of the three ABC transporters at the mRNA (whole genome gene expression array) and/or the protein level (Western Blotting) had been determined previously. In SW480 and U2-OS cells, ABCB1 was expressed at both, the mRNA and the protein level. In U2-OS cells, ABCB1 was found to be overexpressed (Figure 4A). Moderate *ABCB1* mRNA and ABCB1 levels were determined in HCT116 cells.

In the cell lines DMS114, HCC827, HCT116, SW480, A2780 and U2-OS, *ABCC1* was found to be expressed at the mRNA level (Figure 4B). With the exception of HCC827, mRNA expression was accompanied with (moderate) expression of ABCC1. In the cell lines A549, NCI-H520, SW1573, KB-3-1 and MG-63, ABCC1 was expressed at the protein level (expression at the mRNA level was not determined).

Only few cell lines (DMS114, A549, HCC827 and MCF-7) were found to express ABCG2 (Figure 4C). In HCC827 cells, low levels of *ABCG2* mRNA were detected.

### Promoter methylation patterns of *ABCB1*, *ABCC1* and *ABCG2* in MDR cell models

The promoter methylation status of *ABCB1*, *ABCC1* and *ABCG2* (Figure 1) was determined in GLC-4/adr, an adriamycin-resistant subline of GLC-4, two adriamycin-resistant sublines of SW1573, namely SW1573/2R120 and SW1573/2R160, two drug-resistant sublines of KB-3-1, KBC-1 (selected against colchicine) and KB-1089 (selected against the thiosemicarbazone KP1089) and two drug-resistant sublines of HL-60, HL-60/adr (adriamycin-selected) and HL-60/vinc (vincristine-selected). In addition, we analyzed GLC-4/rev, an adriamycin-revertant subline of GLC-4/adr.

**Figure 5:**
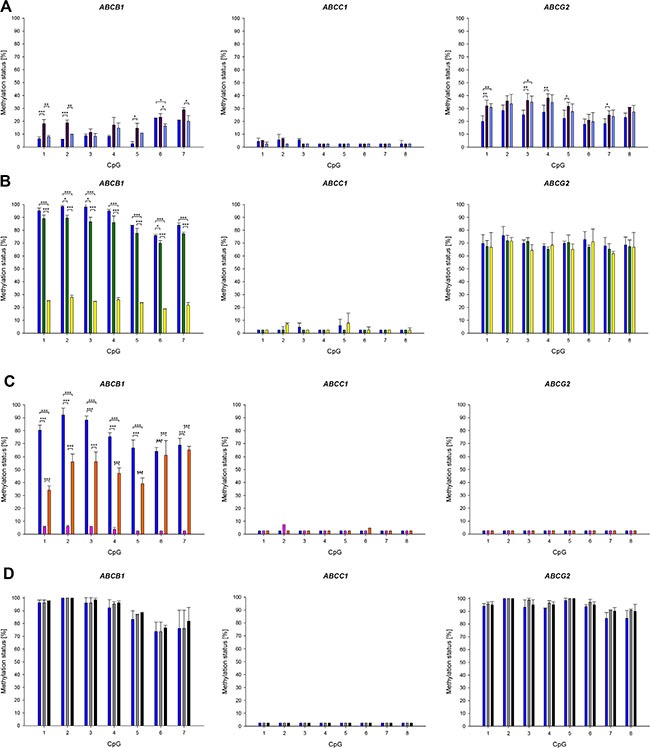
Promoter methylation patterns of *ABCB1, ABCC1* and *ABCG2* in parental cell lines and their drug-resistant sublines (**A**) GLC-4 (dark blue), GLC-4/adr (purple) and GLC-4/rev (light blue). (**B**) SW1573 (dark blue), SW1573/2R120 (green) and SW1573/2R160 (yellow). (**C**) KB-3-1 (dark blue), KB-C-1 (pink) and KB-1089 (orange). (**D**) HL-60 (dark blue), HL-60/adr (grey) and HL-60/vinc (black). Mean and standard deviation of at least four technical replicates. *p < 0.05, **p ≤ 0.01, ***p ≤ 0.001.

No significant difference was found in the extent of *ABCC1* promoter methylation between GLC-4, GLC-4/adr and GLC-4/rev cells, with all three cell lines showing an average methylation status below or slightly higher than 5% (Figure 5A). In contrast, the seven CpGs in the *ABCB1* promoter showed a methylation status ≥ LOQ in GLC- 4, GLC-4/adr and GLC-4/rev cells (with the exception of CpG5 in GLC-4 cells). In the adriamycin-resistant subline, CpG1, CpG2 and CpG5 were significantly higher methylated than in the parental cell line, whereas in the revertant subline, CpG1, CpG2, CpG6 and CpG7 were significantly lower methylated than in GLC-4/adr cells. With the exception of CpG6, no significant difference was found between the *ABCB1* promoter methylation status in GLC-4/rev and the parental cells. In the *ABCG2* promoter, CpG1, CpG3, CpG4, CpG5 and CpG7 were significantly higher methylated in GLC-4/adr than in GLC-4 cells. In contrast to *ABCB1*, no significant difference was found in the *ABCG2* promoter methylation status between GLC-4/adr and GLC-4/rev cells. However, for CpG1 and CpG3 significant differences between GLC-4/rev and GLC-4 cells were detected. In addition to the DNA methylation changes observed for *ABCB1* and *ABCG2*, selection of GLC-4 cells for resistance to adriamycin was associated with a highly significant decrease (from 89 to 65%, *p* < 0.001) in the DNA methylation status of LINE-1 (long interspersed nuclear element 1), a surrogate marker of global DNA methylation (data not shown). No significant difference was found between LINE-1 methylation in GLC-4/adr and GLC-4/rev cells.

In accordance to the results described above, the *ABCC1* promoter was hypomethylated (average methylation status < LOQ) in the parental cell line SW1573 and its drug-resistant sublines SW1573/2R120 and SW1573/2R160 (Figure 5B). In SW1573/2R160, the *ABCB1* promoter was significantly lower methylated than in the parental cell line. A lower methylation status was also observed for SW1573/2R120, in CpG2, CpG3 and CpG6 the difference (compared to the parental cell line) was statistically significant. No significant difference was found in the methylation status of *ABCG2* between the parental cell line and its two adriamycin-resistant sublines.

In the cell line KB-3-1 and its drug-resistant sublines KBC-1 and KB-1089 (Figure 5C), the promoters of *ABCC1* und *ABCG2* were unmethylated (average methylation status < LOQ). In contrast, a difference was found in the promoter methylation status of *ABCB1*. In KB-3-1 cells, the seven CpGs investigated showed a methylation status ≥ 65%, whereas in KBC-1 the methylation status was ≤ 6%. In KB-1089 cells, CpG1-CpG5 were significantly lower methylated than in the parental cell line.

In HL-60 and its drug-resistant cell lines HL-60/adr and HL-60/vinc, the promoters of *ABCB1* and *ABCG2* were highly methylated, whereas that of *ABCC1* was found to be unmethylated (< LOQ) (Figure 5D). No difference was detected between the parental cell line and the drug-resistant sublines.

### Copy number variation of *ABCB1*, *ABCC1* and *ABCG2* in MDR cell models

**Figure 6:**
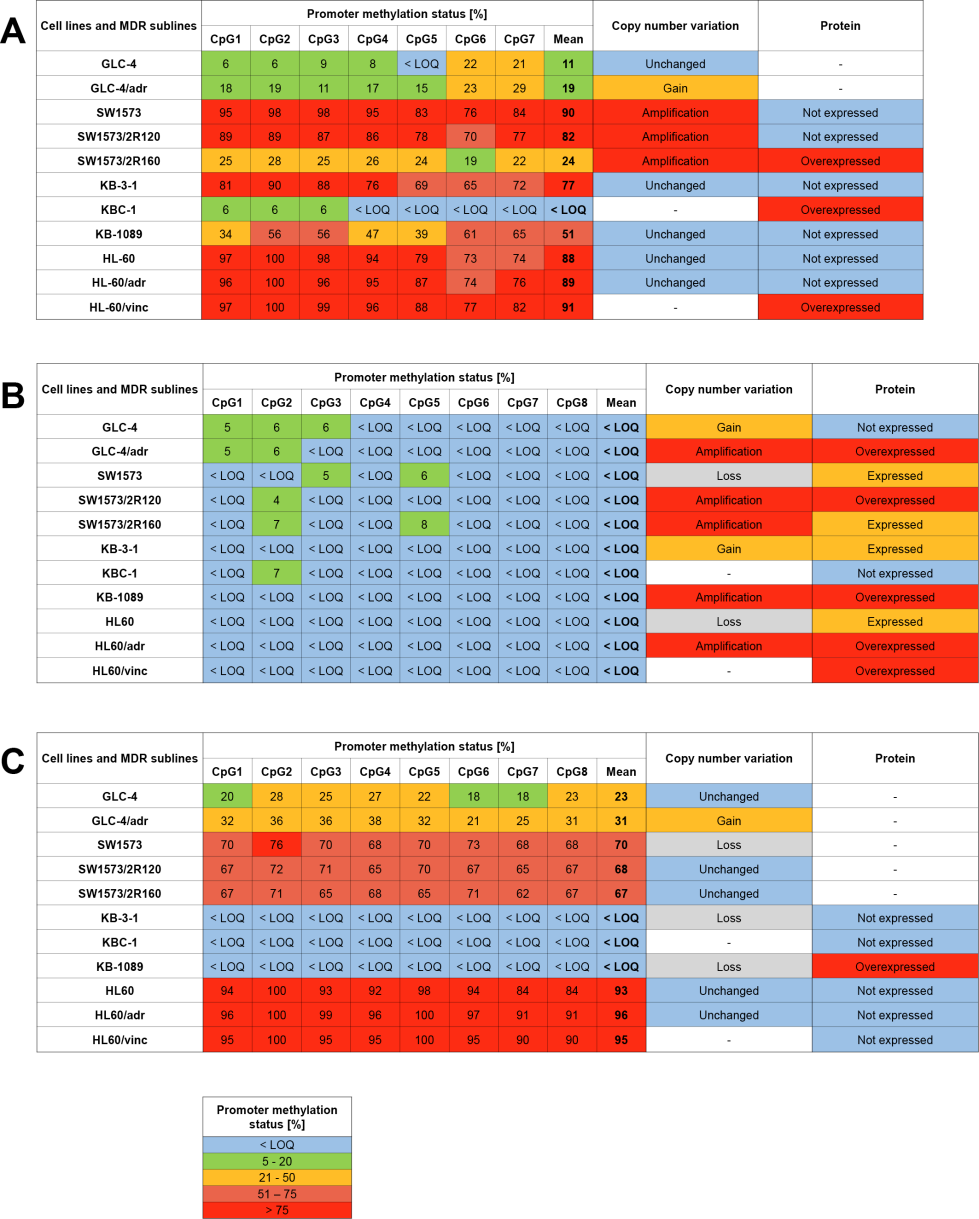
Association of promoter methylation status, copy number variation and expression of *ABCB1* (A), *ABCC1* (B) and *ABCG2* (C) in MDR cell models Limit of quantification (LOQ): 5%. “-” not determined.

Compared to GLC-4, GLC-4/adr showed gene amplification of *ABCC1* as well as a gain in copy numbers of *ABCB1* and *ABCG2* (Figure 6). *ABCB1* and *ABCC1* were amplified in both drug-resistant cell lines of SW1573, SW1573/2R120 and SW1573/2R160. However, *ABCB1* was also amplified in the parental cell line. In addition, gene amplification of *ABCC1* was detected in the drug-resistant cell lines KB-1089 and HL-60/adr, compared to their parental cell lines KB-3-1 and HL-60, respectively.

### Expression of ABCB1, ABCC1 and ABCG2 in MDR cell models

In Western Blot analysis, GLC-4/adr, the adriamycin-resistant subline of GLC-4, showed overexpression of ABCC1 (Figure 6). Compared to the parental cell line SW1573, ABCB1 and ABCC1 were found to be upregulated in SW1573/2R160 and SW1573/2R120 cells, respectively. KBC-1 but not KB-1089 cells showed higher expression of ABCB1 than the parental cell line KB-3-1. KB-1089 cells, however, overexpressed ABCC1 and ABCG2. In the drug-resistant sublines HL-60/vinc and HL-60/adr, higher ABCC1 levels were detected than in the parental cell line HL-60. In addition, HL-60/vinc overexpressed ABCB1.

### Promoter methylation patterns of *ABCB1*, *ABCC1* and *ABCG2* in tumor, tumor-adjacent and tumor-distant tissues from breast cancer patients and normal breast tissues from the healthy control group

Our three bisulfite PSQ methods were applied to determine the promoter methylation levels of *ABCB1*, *ABCC1* and *ABCG2* (Figure 1) in biopsy samples from 16 breast cancer patients. From each patient, three tissue specimens were analyzed: tumor tissue, tumor-adjacent tissue (about 1 cm distance to the tumor) and tumor-distant tissue (about 3 cm distance to the tumor).

**Figure 7:**
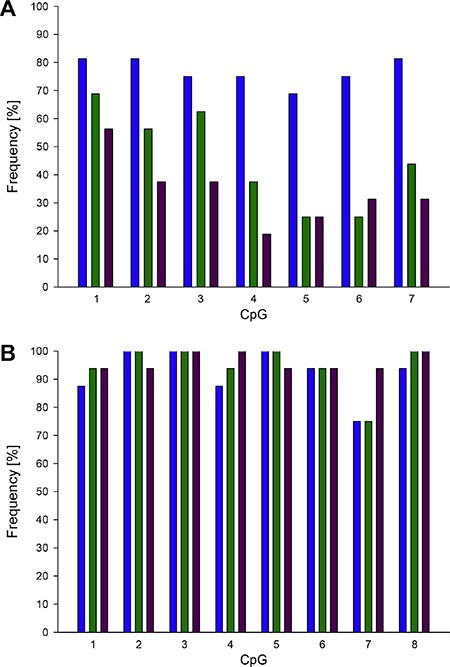
Frequency of promoter methylation of *ABCB1* (A) and *ABCG2* (B) in breast cancer patients Blue: tumor tissue, green: tumor-adjacent tissue, purple: tumor-distant tissue. Limit of quantification (LOQ): 5%.

**Figure 8:**
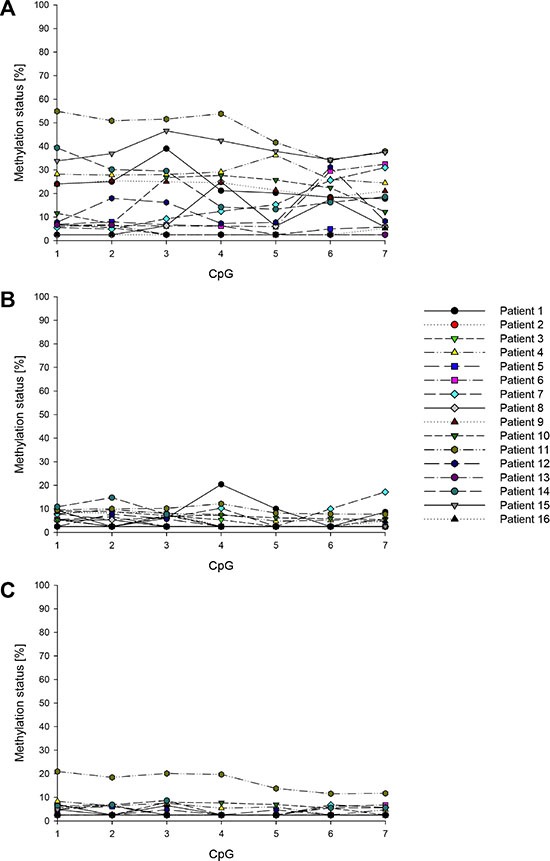
Promoter methylation status of *ABCB1* in breast cancer patients (**A**) Tumor tissue, (**B**) tumor-adjacent tissue, (**C**) tumor-distant tissue. Mean values of at least two technical replicates.

In each of the tumor, tumor-adjacent and tumor-distant tissues as well as in breast tissues from healthy women, the *ABCC1* promoter was hypomethylated (methylation status < LOQ). The seven CpGs in the *ABCB1* promoter were found to be methylated (methylation status ≥ 5%) in at least eleven of the 16 tumor tissues (Figure 7A). In tumor-adjacent and tumor-distant tissues, the target region was less frequently methylated than in tumor tissues. In tumor-distant tissues, CpG1-CpG4 and CpG7 showed less frequently a methylation status ≥ 5% than in tumor-adjacent tissues. CpG1 and CpG2 were methylated in three and CpG4 and CpG6 in one out of four breast tissues from healthy women. In all normal breast tissues, the methylation status of CpG3, CpG5 and CpG7 was < 5%. Figure 8 summarizes the methylation levels of the *ABCB1* promoter in tumor (Figure 8A), tumor-adjacent (Figure 8B) and tumor-distant (Figure 8C) tissues from the 16 breast cancer patients. In tumor tissues, the average methylation status (across the seven CpGs investigated) ranged from < LOQ to 46%. In several patients (patients 1, 6, 7, 8, 10, 12 and 14) the target region was methylated heterogeneously. In all tumor-adjacent and tumor-distant tissues, the average methylation status was found to be ≤ 10%.

**Figure 9:**
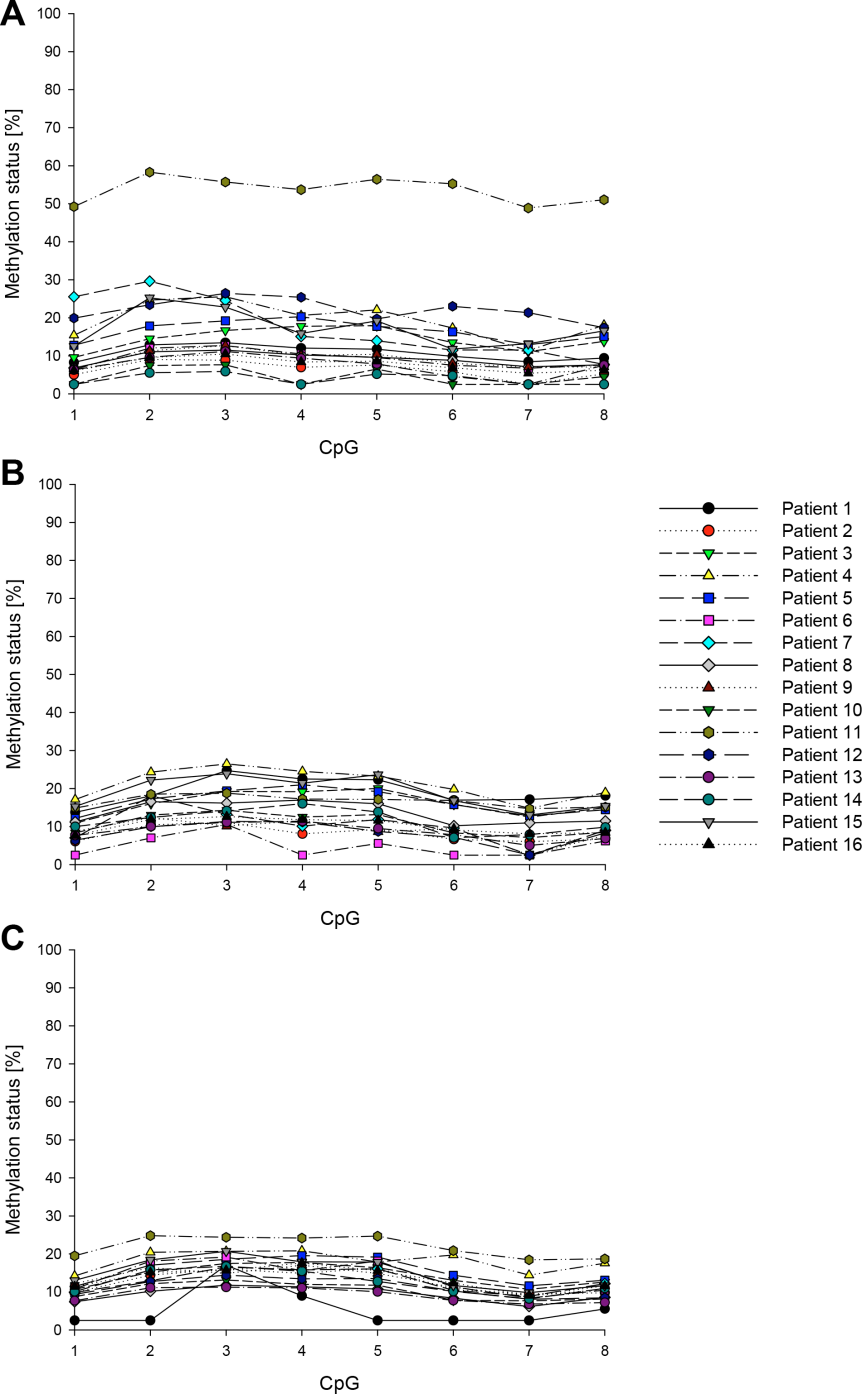
Promoter methylation status of *ABCG2* in breast cancer patients (**A**) Tumor tissue, (**B**) tumor-adjacent tissue, (**C**) tumor-distant tissue. Mean values of at least two technical replicates.

With the exception of CpG7, the CpGs targeted in the *ABCG2* promoter were methylated (methylation status ≥ 5%) in at least 14 out of the 16 tumor, tumor-adjacent and tumor-distant tissues (Figure 7B). In the breast tissues from healthy women, the eight CpGs were also found to be methylated, with the exception of CpG7 in one tissue. The methylation levels of *ABCG2* obtained for tumor, tumor-adjacent and tumor-distant tissues are summarized in Figure 9. In general, the *ABCG2* promoter was methylated more homogenously than the promoter of *ABCB1*. In accordance to the results obtained for *ABCB1*, tumor tissue of patient 11 showed the highest methylation status (average methylation status 54%). In all other tumor tissues, the average methylation status was ≤ 22%. In most tumor-adjacent and tumor-distant tissues, the methylation status was ≤ 20%.

**Figure 10:**
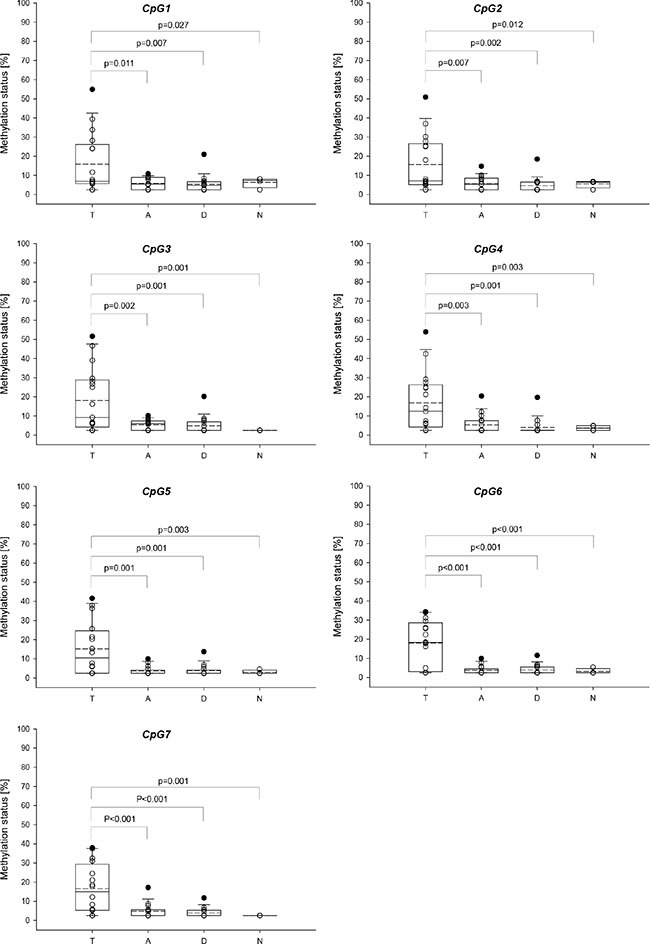
Distribution of the promoter methylation status of *ABCB1* in breast cancer patients and healthy controls T: Tumor tissue, A: tumor-adjacent tissue, D: tumor-distant tissue, N: normal breast tissue. Straight line: median, dashed line: arithmetic mean.

**Figure 11:**
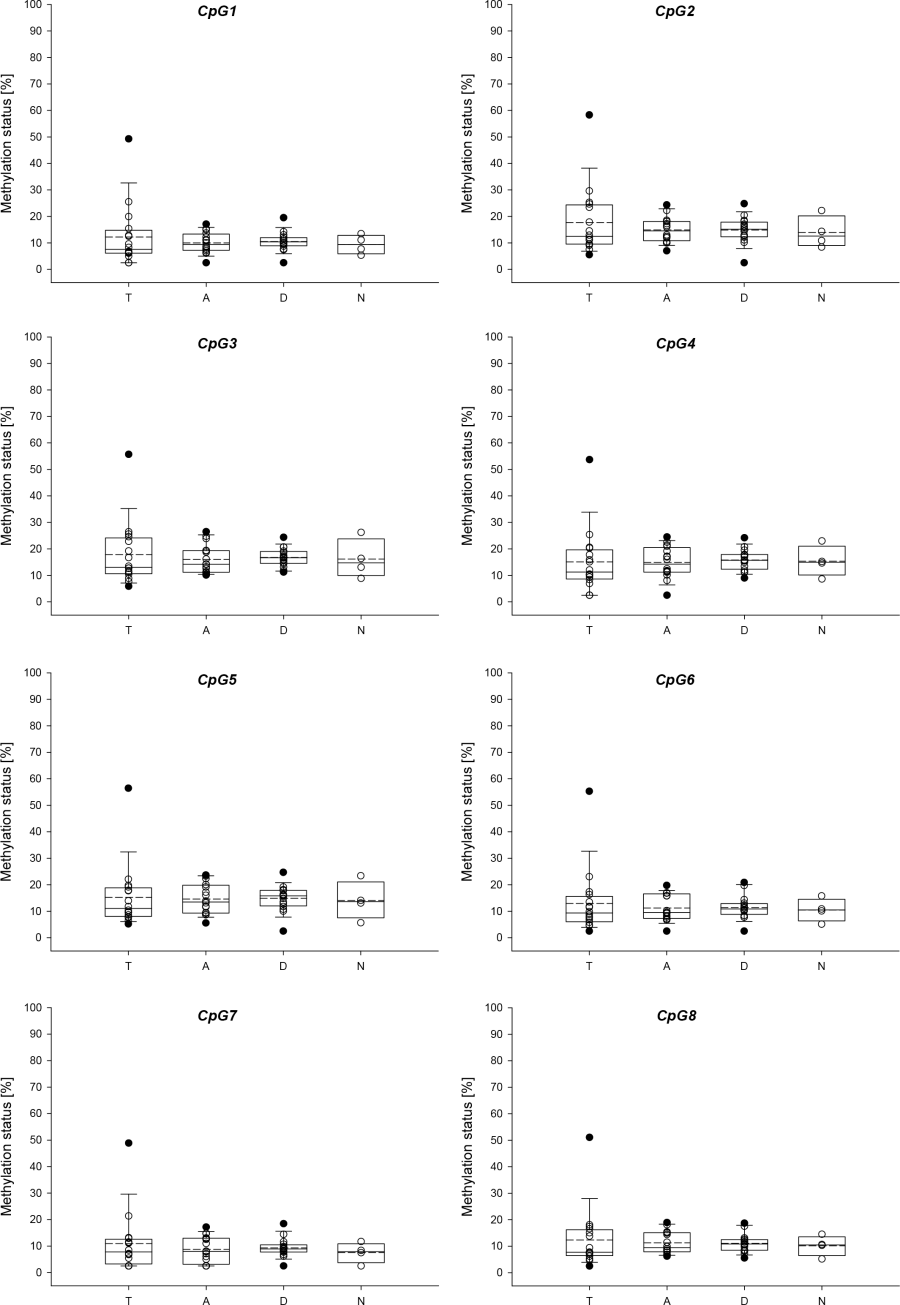
Distribution of the promoter methylation status of *ABCG2* in breast cancer patients and healthy controls T: Tumor tissue, A: tumor-adjacent tissue, D: tumor-distant tissue, N: normal breast tissue. Straight line: median, dashed line: arithmetic mean.

The distribution of the methylation levels in tumor, tumor-adjacent and tumor-distant tissues and breast tissues from healthy women for *ABCB1* and *ABCG2* is shown in Figure 10 and Figure 11, respectively. For each of the seven CpGs in the *ABCB1* promoter, a statistically significant difference was found between tumor and tumor-adjacent tissues, tumor and tumor-distant tissues as well as between tumor and normal breast tissues of the control group (Figure 10). However, no difference was found between tumor-adjacent and tumor-distant tissues. In addition, no difference was found in the methylation status of the *ABCG2* promoter between tumor, tumor-adjacent and tumor-distant tissues and breast tissues from healthy women(Figure 11).

### Correlation between the promoter methylation levels of different genes

Statistical analyses revealed that in tumor tissues the average promoter methylation status of *ABCB1* (across the seven CpGs investigated) significantly correlated with the average promoter methylation status of *ABCG2* (across the eight CpGs investigated) (*r* = 0.621; *p* = 0.010). In a previous study, we have determined the promoter methylation status of six tumor suppressor genes, comprising *CCND2* (cyclin D2), *DAPK1* (death-associated protein kinase 1), *GSTP1* (glutathione S-transferase P1), *HIN-1* (high in normal-1), *MGMT* (O6-methylguanine-DNA methyltransferase) and *RASSF1A* (Ras association domain family member 1), in tumor, tumor-adjacent and tumor-distant tissues from the same breast cancer patients by methylation-sensitive high resolution melting analysis [45]. We did not find a statistically significant correlation between the average methylation status of the ABC transporters and that of *CCND2, DAPK1, GSTP1, HIN-1, MGMT* or *RASSF1A*.

### Association of the promoter methylation status of *ABCB1*, *ABCC1* and *ABCG2* with clinicopathological parameters

The methylation status of CpG3-CpG7 of the *ABCB1* promoter in tumor tissues was associated with the menopause status of the patients (*p* = 0.008, *p* = 0.008, *p* = 0.002, *p* = 0.008 and *p* = 0.017, respectively). In patients with post-menopausal status the methylation levels of these CpGs were ≥ LOQ, whereas in patients with pre-menopausal status the methylation levels were < LOQ. Methylation levels of CpG6 and CpG7 in tumor tissues were found to correlate with the age of the patients (*r* = 0.674, *p* = 0.004 and *r* = 0.688, *p* = 0.003, respectively). The methylation status of CpG7 in tumor-adjacent tissues was significantly associated with tumor grading (*p* = 0.017). In patients with tumor grade 3, the methylation status was < LOQ, whereas in patients with tumor grade 1 and 2, the methylation status was found to be ≥ LOQ. In addition, a correlation was found between the methylation status of CpG6 and CpG7 in the *ABCG2* promoter in tumor-distant tissues and MIB-1 (*r* = 0.556, *p* = 0.025 and *r* = 0.550, *p* = 0.027, respectively).

## DISCUSSION

In the present study, we determined the promoter methylation patterns of *ABCB1*, *ABCC1* and *ABCG2* in human cancer cell lines, MDR cell models and tumor, tumor-adjacent and tumor-distant tissues from breast cancer patients. Various technologies allow determining the methylation status of candidate genes, with each of them having strengths and limitations. We applied bisulfite pyrosequencing (PSQ) because in contrast to other common technologies, e.g. methylation-specific polymerase chain reaction (MS-PCR), MethyLight and methylation-sensitive high resolution melting (MS-HRM), bisulfite PSQ makes determination of the methylation status of individual CpGs possible. Compared to bisulfite sequencing, the gold standard of DNA methylation analysis, bisulfite PSQ is less costly in terms of money, labor and time. The bisulfite PSQ methods applied were developed in-house. Primers for *ABCB1*, *ABCC1* and *ABCG2* were designed to target CpG-rich fragments of the respective promoters. We were interested in the DNA methylation status of the main promoters known to regulate most of the transcriptional activity but not in that of alternative promoters linked to tissue-specific gene expression [20, 46]. The method for *ABCB1* is applicable to determine the methylation status of seven CpGs downstream of the TSS, the methods for *ABCC1* and *ABCG2* target eight CpGs upstream of the respective TSS (Figure 1). The functional relevance of the DNA methylation status of the seven CpGs in the *ABCB1* promoter has been discovered recently. A significant difference was found in the DNA methylation status of these CpGs between parental SW480 cells and SW480/tria, a triapine-resistant subline overexpressing ABCB1 [47]. In addition, Reed *et al.* reported significant hypomethylation of this region in drug-resistant sublines of MCF-7 compared to the parental breast cancer cell line [28]. The sequence to analyze of the *ABCB1* promoter has been predicted to contain binding sites for glucocorticoid receptor-alpha (GR-α), yin-jang 1 (YY1) and general transcription factor II-I (TFII-I) [48]. YY1, involved in regulation of gene transcription and protein modifications, is assumed to play a proliferative or oncogenic role in carcinogenesis [49], whereas TFII-I is known to be a mediator of growth factor signalling. The eight CpGs targeted by our *ABCC1* method are directly upstream of the CpGs that have been found to be hypomethylated in the pancreatic cancer cell line SW1990 and its drug-resistant subline SW1990/GZ [30]. The sequence to analyze contains a putative binding site for paired box 5 (Pax5) [48], which is assumed to play an oncogenic role in B cell malignancies [50]. The eight CpGs in the *ABCG2* promoter are overlapping with the CpGs targeted by To *et al*. [41] or in close distance to the regions investigated in previous studies [29, 31, 42]. The sequence to analyze has been predicted to contain a binding site for c-jun, a well-known proto-oncogene [48].

The 19 human cancer cell lines investigated in the present study were found to differ in the methylation status of the *ABCB1* and *ABCG2* promoter. However, in each of the cell lines, the *ABCC1* promoter was found to be unmethylated (average methylation status across eight CpGs ≤ 5%). In most cell lines, the *ABCB1* promoter was higher methylated than the *ABCG2* promoter, with the exception of the two small cell lung cancer cell lines GLC-4 and DMS114, the non-small cell lung cancer cell line A549 and the prostate cancer cell line PC-3.

Among the small cell lung cancer cell lines, in GLC-4 cells the promoters of both *ABCB1* and *ABCG2* were found to be higher methylated than in DMS114 cells.

Quite different methylation levels were obtained for the five non-small cell lung carcinoma cell lines investigated. In two cell lines, SW1573 and NCI-H1703, both the *ABCB1* and *ABCG2* promoter showed a methylation status in the range from 70 to 90%. In A549 and NCI-H520 cells, the average methylation status of the *ABCB1* and *ABCG2* promoter was ≤ 20% and 32%, respectively. In contrast, in HCC827 cells, the *ABCB1* promoter was found to be highly methylated (average methylation status 78%), whereas the *ABCG2* promoter only showed an average methylation status of 5%. The histological type of the lung cancer (adenocarcinoma: A549, HCC827; squamous: SW1573, NCI-H520, NCI-H1703) was not found to have an impact on the methylation status of the *ABCB1* and *ABCG2* promoters. Our results are in accordance with a previous study pointing to differences in the *ABCB1* promoter methylation status between various lung cancer cell lines [43]. In the study of Bram *et al*. [29], in A549 cells the *ABCG2* promoter (CpGs from −380 to +6 relative to the TSS) has been found to be devoid of methylation which is a discrepancy to our results.

In the two colon cancer cell lines, HCT116 and SW480, the average methylation status of *ABCB1* was about 40%. In SW480 cells the *ABCG2* promoter was higher methylated (18%) than in HCT116 cells (< 5%). *ABCB1* promoter methylation in HCT116 cells has already been reported previously [51].

In all three breast cancer cell lines investigated, the *ABCB1* promoter was found to be highly methylated. The *ABCG2* promoter was methylated (average methylation status 13–21%) in the estrogen receptor-positive cell lines MCF-7 (HER2-negative, Luminal A) and ZR-75-1 (HER2-positive, Luminal B), but was < LOQ in the triple negative cell line MDA-MB-231. Hypermethylation of the *ABCB1* promoter (CpGs from −164 to about +600 relative to the TSS) in MCF-7 cells has already been reported by David *et al*. [27]. Bram *et al*. [29] have found MCF-7 cells to be devoid of *ABCG2* promoter methylation (CpGs from −380 to +6 relative to the TSS).

In the prostate cancer cell line PC-3, both the *ABCB1* (79%) and the *ABCG2* (97%) promoter were highly methylated. Hypermethylation of the *ABCB1* promoter has already been reported for several prostate cancer cell lines including PC-3 [37], DU145 and ND1 cells [32]. In both, the cervix cancer cell line KB-3-1 and the ovarian carcinoma cell line A2780, the *ABCB1* promoter was found to be highly methylated, whereas the *ABCG2* promoter was unmethylated. In both osteosarcoma cell lines investigated, promoter methylation levels of *ABCB1* and *ABCG2* were in the range from 10 to 17%. In the multiple myeloma cell line U266, the *ABCB1* promoter was found to be slightly methylated, that of *ABCG2* was < LOQ. In the promyelocytic leukemia HL-60, the promoters of *ABCB1* and *ABCG2* were highly methylated. Extensive *ABCB1* promoter methylation in HL-60 cells has been described previously [52].

Results obtained by whole genome gene expression array and Western Blot analyses indicate that in the cancer cell lines investigated, the promoter methylation status of *ABCC1* is not associated with mRNA and/or protein expression levels. Although in general, the eight CpGs were unmethylated (methylation status < LOQ), some cancer cell lines (e.g. DMS114, NCI-H520 and SW1573) expressed, whereas others (e.g. GLC-4, MCF-7 and HL- 60) did not express ABCC1.

In cancer cell lines with (weak) expression of ABCG2 (DMS114, A549, HCC827 and MCF-7), the mean promoter methylation status of *ABCG2* was in the range from 5 to 32%. Although in e.g. NCI-H520, HCT116 and KB-3-1 cells, the mean promoter methylation status of *ABCG2* was also very low, ABCG2 expression was, however, not detected. Thus, the promoter methylation status of the eight CpGs targeted by PSQ is not assumed to be involved in gene regulation of *ABCG2*.

In contrast, in some cancer cell lines, promoter methylation of *ABCB1* was found to be inversely correlated with gene expression at the mRNA and/or protein level. The cancer cell lines HCC827, SW1573, KB-3-1 and HL-60, which showed high promoter methylation status of *ABCB1*, did not express ABCB1. In HCT116, SW480 and U2-OS cells, displaying low to moderate methylation status of the *ABCB1* promoter, *ABCB1* was found to be expressed at both the mRNA and protein level. This finding indicates that promoter methylation is an important mechanism in gene regulation of *ABCB1*.

Copy number variation analyses revealed that the copy numbers of *ABCB1*, *ABCC1* and *ABCG2* were not associated with the promoter methylation levels of the three ABC transporters.

Currently, numerous studies are carried out attempting to overcome MDR in cancer. The data on the methylation patterns of *ABCB1*, *ABCC1* and *ABCG2* provided in the present study will help in selecting appropriate cancer cell lines for investigating the mode of action and/or testing the efficacy of (epigenetic) anticancer drugs.

In order to further elucidate the role of DNA methylation changes in acquisition of a MDR phenotype, we determined the methylation patterns of *ABCB1*, *ABCC1* and *ABCG2* in several MDR cell models. In GLC-4/adr and GLC-4/rev, an adriamycin-revertant subline of GLC-4/adr, the *ABCC1* promoter showed a methylation status < LOQ, independent of the ABCC1 expression level in the cells. Results obtained by array CGH indicate that overexpression of *ABCC1* in GLC-4/adr is mediated by gene amplification. In GLC-4/adr cells, both the *ABCB1* and *ABCG2* promoter were higher methylated than in the parental cell line. The difference in the methylation status was statistically significant in three and five CpGs, respectively. In addition, in GLC-4/adr cells we observed significant lower methylation of LINE-1 compared to the parental cell line. Our results indicate that acquisition of the MDR phenotype was associated with a substantial decrease in global DNA methylation. Methylation levels determined for GLC-4/rev suggest that changes in the promoter methylation status of *ABCB1* were, at least in part, reversed by culturing the MDR subline without selection pressure.

In the parental cell line SW1573 and its drug-resistant sublines SW1573/2R120 and SW1573/2R160, the *ABCC1* promoter was found to be unmethylated (average methylation status < 5%), independent of the expression level of ABCC1. Array CGH analysis showed that the (over)expression of ABCC1 in SW1573/2R120 and SW1573/2R160 cells is based on gene amplification. As expected, in the ABCB1- and ABCC1-overexpressing subline SW1573/2R160, the *ABCB1* promoter was significantly lower methylated than in the parental cell line. Gene amplification of *ABCB1* was observed for the two adriamycin-resistant sublines, but also for the parental cell line. For *ABCG2*, no significant difference was found between the parental cell line and its two adriamycin-resistant sublines, neither in the methylation status, nor in the expression levels or the gene copy numbers.

In KB-3-1 and its drug-resistant sublines KBC-1 and KB-1089, the promoters of *ABCC1* and *ABCG2* were unmethylated (methylation status < LOQ). These results indicate that overexpression of ABCC1 and ABCG2 in KB-1089 cells is not mediated by DNA methylation. Array CGH analysis revealed that overexpression of ABCC1 is due to gene amplification. However, overexpression of ABCG2 seems to be mediated by an alternative mechanism. Overexpression of ABCB1 in KBC-1 cells is accompanied by a substantial decrease in the promoter methylation status of *ABCB1*. Although KB-1089 cells were not found to express ABCB1, CpG1-CpG5 were significantly lower methylated than in the parental cell line.

For none of the ABC transporters, a significant difference in the methylation status was found between HL-60 and its two drug-resistant sublines HL-60/vinc and HL-60/adr, although HL-60/vinc was found to overexpress ABCB1 and ABCC1 and HL-60/adr overexpresses ABCC1. Overexpression of ABCC1 in HL-60/adr was associated with amplification of *ABCC1*.

Our results are in accordance with previous studies reporting that the overexpression of ABCB1 in MDR cancer cells is mediated by a decrease in *ABCB1* promoter methylation [26, 27]. Our data on *ABCC1* is consistent with that of Chen *et al.* [30] who have not found a difference in *ABCC1* promoter methylation between the pancreatic cancer cell line SW1990 and its ABCC1-overexpressing subline SW1990/GZ.

A few papers indicate that hypermethylation of the *ABCB1* promoter is a frequent event in breast cancer [34, 36, 38], so far data on promoter methylation of *ABCC1* and *ABCG2* has, however, not been published. Since changes in the DNA methylation status are known to be an early event in carcinogenesis, we analyzed tumor, tumor-adjacent and tumor-distant tissues from the same breast cancer patients to find out if aberrant DNA methylation levels even occur in tissues that appear histologically normal. In contrast to the *ABCC1* promoter which was found to be hypomethylated (methylation status < LOQ) in all tumor, tumor-adjacent and tumor-distant tissues, the *ABCB1* and *ABCG2* promoters were found to be methylated very frequently. Each of the CpGs in the *ABCB1* and *ABCG2* promoters was methylated in ≥ 75% of the tumor tissues. In a previous study, *ABCB1* promoter methylation has been detected in 39.3% of 28 small invasive ductal carcinomas [36]. By investigating several regions of the *ABCB1* promoter, Dejeux *et al.* [34] have observed methylation in 64 – 81% of locally advanced breast tumors. Klajic *et al.* [38] have reported *ABCB1* promoter methylation in 47.6% (invasive stage I tumors) to 70% (invasive stage IV tumors) of a total of 238 breast cancer tissue samples. In contrast to our results and those published by Muggerud *et al.* [36] and Klajic *et al.* [38], Sharma *et al.* [35] have found the *ABCB1* promoter to be hypomethylated in 47% of tumors from 100 invasive ductal breast carcinoma patients. In the present study, the *ABCB1* promoter was more frequently methylated in tumor tissues than in tumor-adjacent and tumor-distant tissues, whereas in case of the *ABCG2* promoter, no difference was found between the three tissue specimens. In each of the breast tissues from the control group, the *ABCC1* promoter showed an average methylation status < 5%. In three of four normal breast tissues from healthy women, the *ABCB1* promoter was unmethylated, whereas the *ABCG2* promoter was found to be methylated (average methylation status from 6 to 19%) in all breast tissues from the control group. Our data on *ABCB1* promoter methylation in normal breast tissues is consistent with the study of Muggerud *et al.* [36] reporting absence of DNA methylation around the TSS in normal breast tissue.

In the tumor tissues analyzed in the present study, the average *ABCB1* promoter methylation status ranged from < LOQ to about 50%, whereas in most tumor-adjacent and tumor-distant tissues, the average methylation status was ≤ 10%. In case of *ABCG2*, all tumor tissues except one showed an average methylation status < 25%. In most tumor-adjacent and tumor-distant tissues, the average methylation status was ≤ 22%. Strikingly, the highest methylation status of both the *ABCB1* and *ABCG2* promoter was obtained for a triple negative breast tumor. In addition to the triple negative status, the tumor showed a high proliferative activity (MIB-1: 70%). However, since it was the only triple negative tumor sample analyzed in the present study, we cannot draw any conclusions from our result. As described above, in the triple negative cell line MDA-MB-231 the *ABCB1* promoter was highly methylated. However, the *ABCG2* promoter was unmethylated (average methylation status < LOQ). In a previous study, for estrogen receptor-positive tumors higher *ABCB1* promoter methylation levels have been reported than for estrogen receptor-negative ones [36]. In accordance with the study of Muggerud *et al.* [36], Dejeux *et al.* [34] have observed a trend for the absence of *ABCB1* promoter methylation in basal-like breast tumors.

For each of the seven CpGs in the *ABCB1* promoter investigated, a statistically significant difference was found between tumor and tumor-adjacent tissue, tumor and tumor-distant tissue as well as between tumor and normal breast tissues of the control group. However, no difference was found between tumor-adjacent and tumor-distant tissues. In addition, no difference was found in the methylation status of the *ABCG2* promoter between tumor, tumor-adjacent, tumor-distant tissues and breast tissues from healthy women. These results indicate that neither the *ABCB1* nor the *ABCG2* promoter methylation status is applicable as indicator for detecting field cancerization in breast cancer.

Statistical analyses revealed that in tumor tissues the average promoter methylation status of *ABCB1* significantly correlated with that of *ABCG2*. However, we did not find a correlation between the methylation status of *ABCB1* or *ABCG2* and that of *CCND2, DAPK1, GSTP1, HIN-1, MGMT* or *RASSF1A* determined previously [45]. In patients with post-menopausal status, the methylation levels of CpG3-CpG7 in the *ABCB1* promoter were significantly higher than in patients with pre-menopausal status. In addition, methylation levels of CpG6 and CpG7 were found to positively correlate with the age of the patients. Tumor-adjacent tissues from patients with tumor grade 3 showed significantly lower methylation status of CpG7 than tumor-adjacent tissues from patients with tumor grade 1 or 2. In addition, positive correlations were found between MIB-1 and the methylation status of CpG6 and CpG7 in the *ABCG2* promoter in the tumor-distant tissues. In a previous study, lower *ABCB1* promoter methylation levels have been reported for Ki67-positive tumors than for Ki67-negative ones [36].

In conclusion, in-house developed bisulfite PSQ methods were applied to determine the promoter methylation status of *ABCB1*, *ABCC1* and *ABCG2* in 19 human cancer cell lines, MDR cell models as well as tumor, tumor-adjacent and tumor-distant tissues from 16 breast cancer patients. In all cancer cell lines, the *ABCC1* promoter was found to be unmethylated. However, the cancer cell lines showed substantial differences in the promoter methylation status of the *ABCB1* and *ABCG2* promoters. In some cancer cell lines, promoter methylation of *ABCB1* was found to be inversely correlated with gene expression at the mRNA and/or protein levels, indicating, that promoter methylation is an important mechanism in gene regulation of *ABCB1*. Analysis of MDR cell models revealed that overexpression of ABCB1 is linked to a decrease in *ABCB1* promoter methylation, whereas upregulation of ABCC1 was frequently mediated by gene amplification. The *ABCC1* promoter was found to be hypomethylated in all tumor, tumor-adjacent and tumor-distant tissues from breast cancer patients as well as in breast tissues from healthy women. In contrast, the promoters of *ABCB1* and *ABCG2* were found to be methylated in ≥ 75% of the tumor tissues. The *ABCB1* promoter was more frequently methylated in tumor tissues than in tumor-adjacent and tumor-distant tissues, whereas for the *ABCG2* promoter, no difference was found between the three tissue specimens. Statistically significant differences were found in the *ABCB1* promoter methylation status between tumor and tumor-adjacent tissue, tumor and tumor-distant tissue as well as between tumor and normal breast tissues from the control group.

## MATERIALS AND METHODS

### Cell lines

The following 19 human cancer cell lines were used in this study: small cell lung carcinoma cell lines DMS114 and GLC-4; non-small cell lung carcinoma cell lines A549, HCC827, NCI-H520, NCI-H1703 and SW1573; colorectal adenocarcinoma cell lines HCT116 and SW480; breast adenocarcinoma cell lines MCF-7 and MDA-MB-231; breast ductal carcinoma cell line ZR-75-1 derived from metastatic site (ascites); epidermal cervical cancer cell line KB-3-1; ovarian carcinoma cell line A2780; prostate cancer cell line PC-3; osteosarcoma cell lines MG-63 and U2-OS; multiple myeloma cell line U266; and promyelocytic leukemia cell line HL-60. The study also included the following drug-resistant sublines: GLC-4/adr (and its revertant subline GLC-4/rev), SW1573/2R120, SW1573/2R160, KBC-1, KB-1089, HL-60/adr and HL-60/vinc. These cancer cell lines were selected because they were available in our lab and originated from a broad variety of tissue types. All details (tissue origin, disease, growth medium and source) regarding the different human cancer cell lines used in this study are shown in Supplementary Table S1. Culture media were supplemented with 10% fetal calf serum (PAA, Austria). Cell cultures were periodically checked for mycoplasma contamination. Cell line authentication was performed by array CGH and/or short tandem repeat fingerprint.

### Patients and breast tissue samples

Breast tissue samples were collected from 16 breast cancer patients at diagnosis. The study was approved by the Ethics Commission of the Medical University of Vienna (application number EK 2011/1074). All breast cancer patients gave written informed consent. The age of the patients ranged from 39 to 76 years (mean: 58 years). None of the women had a family history of breast cancer. In addition, none of the patients had received radiotherapy, chemotherapy or hormonal treatment. Table 2 gives information on the characteristics of the breast cancer patients, including age, menopausal status, histologic type, histological grading, B classification, proliferative activity (MIB-1), status of estrogen receptor (ER), progesterone receptor (PR) and human epidermal growth factor receptor 2 (HER2) as well as the molecular subtype. Three biopsy samples were taken from each patient by ultrasound guided needle biopsies. The first biopsy sample was taken directly from the primary breast tumor (“tumor tissue”), the second one from histologically normal tissue located about 1 cm from the tumor (“tumor-adjacent tissue”) and the third one from histologically normal tissue located about 3 cm from the center of the tumor (“tumor-distant tissue”). Non-cancerous breast tissue samples were collected from four women undergoing breast reduction mammoplasty. The age of these women ranged from 44 to 60 years (mean: 53 years). Tissues from breast cancer patients and healthy controls have already been included in a previous study [45]. Biopsy samples were stored in phosphate-buffered saline (PBS) at −80°C until DNA extraction.

**Table 2:** Patient and tumor characteristics

Patient	Age [y]	Menopause status	Histologic type	Histological grading	B classification	MIB-1 [%]	Receptor status			Molecular subtype
							ER	PR	HER2	
1	65	Post	IDC	2	5b	10	+	+	–	Luminal A
2	54	Peri	IDC	3	5b	30	+	–	–	Luminal A
3	39	Pre	IDC	2	5b	40	+	+	+	Luminal B
4	66	Post	IDC	2	5b	60	+	+	–	Luminal A
5	50	Pre	IDC	3	5b	50	+	+	+	Luminal B
6	73	Post	IDC	3	5b	20	+	+	–	Luminal A
7	76	Post	IDC	2	5b	20	+	+	–	Luminal A
8	63	Post	IDC	3	5	30	+	+	–	Luminal A
9	48	Post	IDC	3	5b	20	+	+	+	Luminal B
10	58	Post	IDC	1	5c	20	+	+	+	Luminal B
11	61	Post	IDC	3	5b	70	–	–	–	Triple negative
12	52	Pre	ILC	n.s.	5b	50	+	+	–	Luminal A
13	42	Pre	IDC	3	5b	80	+	–	–	Luminal A
14	67	Post	IDC	3	5b	40	+	+	–	Luminal A
15	61	Post	ILC	2	5b	30	+	+	–	Luminal A
16	41	Pre	Mucinous	2	5b	50	+	+	+	Luminal B

MIB-1: mindbomb E3 ubiquitin protein ligase 1 (proliferative activity), IDC: invasive ductal carcinoma, ILC: invasive lobular carcinoma, ER: estrogen receptor, PR: progesterone receptor, HER2: human epidermal growth factor receptor 2, n.s.: not specified.

When the biopsy samples were drawn, we were solely interested in DNA methylation analysis. Due to the specific sample preparation procedure applied, the samples could not be subjected to gene expression analysis later on.

### DNA methylation analysis by bisulfite pyrosequencing

Genomic DNA was extracted from cell lines and breast tissue samples by using the *QIAamp DNA Mini Kit* (Qiagen, Germany) according to the manufacturer's instruction. The extracted DNA was quantified using a Nanodrop 2000c spectrophotometer (Thermo Scientific, USA). Human control DNA (*CpGenome Universal Methylated DNA* and *EpiTect Control DNA (human), unmethylated*) was obtained from Millipore (USA) and Qiagen, respectively.

DNA extracted from cancer cell line pellets and breast tissue samples as well as human control DNA was converted with sodium bisulfite by using the *EpiTect Fast Bisulfite Conversion Kit* (Qiagen) according to the manufacturer's protocol.

Bisulfite pyrosequencing methods were developed in-house. The nucleotide sequences of *ABCB1* [GenBank: NG_011513.1], *ABCC1* [GenBank: NG_028268.1] and *ABCG2* [GenBank: NG_032067.2] were taken from the National Center for Biotechnology Information (NCBI) database [53]. Promoter regions were identified using the Transcriptional Regulatory Element Database (TRED) [54]. Transcription factor binding sites were predicted on the PROMO site [48] (*ABCB1* and *ABCC1*: dissimilarity margins were set ≤ 0%; *ABCG2*: dissimilarity margin was set ≤ 3%).

Primers were designed with the PyroMark Assay Design Software 2.0.1.15 (Qiagen). For each primer set the annealing temperature (T_a_) and the primer concentrations were optimized. Primer sequences and optimized conditions are summarized in Table 1. Polymerase chain reaction (PCR) was performed using the *PyroMark PCR Kit* (Qiagen). The reaction volume per well was 25 μl, including 12.5 μl PyroMark PCR Master Mix (2×), 2.5 μl CoralLoad Concentrate (10×), forward and reverse primer, RNase-free water and 10 ng of bisulfite converted DNA. Amplification was performed on an iCycler instrument (Bio-Rad, USA) under the following conditions: initial activation step at 95°C for 15 min, 50 cycles: 30 s at 94°C, 30 s at the respective annealing temperature (Table 1), 30 s at 72°C and a final extension at 72°C for 10 min. The identity and purity of the PCR products were determined by randomly loading them onto a 2% agarose gel in 1× TAE buffer. After staining with *GelRed* (Biotium, USA) bands were visualized with an UVT-20 M transilluminator (Herolab, Germany).

Pyrosequencing analyses were performed using the PyroMark Q24 Advanced instrument (Qiagen) and *PyroMark Q24 Advanced CpG Reagents* (Qiagen). Sample preparation was carried out with the PyroMark Q24 Vacuum Workstation (Qiagen). In brief, 15 μl of biotinylated PCR product was mixed with 1 μl Streptavidin Sepharose High Perfomance (GE Healthcare, Germany), 40 μl PyroMark Binding Buffer and 24 μl high-purity water (Milli-Q 18.2 MΩ) and agitated for 10 min on a shaker at 1400 rpm. The double stranded DNA was denatured, washed and finally transferred into a PyroMark Q24 Plate (Qiagen) containing 20 μl of 0.375 μM sequencing primer. The plate was transferred to a pre-heated (80°C) PyroMark Q24 Plate Holder (Qiagen), hold at 80°C for 5 min and then transferred into the instrument. Pyrosequencing data was evaluated with the PyroMark Q24 Advanced software 3.0.0 (Qiagen).

All samples were analyzed at least twice. A no template control was included in each run, serving as control for contamination.

### Array comparative genomic hybridization (array CGH)

Isolation of genomic DNA and array CGH analysis were performed as described in [55] using 4 × 44K whole genome oligonucleotide-based arrays (Agilent, Canada). Labeling and hybridization procedures were performed according to the instructions provided by Agilent using the *SureTag DNA Labeling Kit*. Slides were scanned with a G2505B Micro Array Scanner (Agilent). Feature extraction and data analysis were carried out using the Feature Extraction and Agilent Genomic Workbench software, respectively.

### Whole genome gene expression arrays (mRNA expression arrays)

Isolation of total RNA and whole genome gene expression arrays were performed as described in [55, 56]. Single or dual color experiments were performed according to the instructions provided by Agilent using the *Quick Amp Labeling Kit*. Slides were scanned with a G2505B Micro Array Scanner (Agilent). Feature extraction and data analysis were carried out using the Feature Extraction and GeneSpring software, respectively.

### Gene expression at the protein level

Membrane-enriched protein extracts were prepared, separated by SDS-PAGE (15 μg protein per sample), and transferred onto a polyvinylidene difluoride membrane for Western Blotting as described previously [57]. Primary antibodies used are given in Supplementary Table S2. Secondary, horseradish peroxidase-labeled antibodies against mouse (goat anti-mouse) and rat (goat anti-rat) were purchased from Santa Cruz Biotechnology and Thermo Scientific, respectively, and used in working dilutions of 1:10 000.

### Statistical analysis

Statistical analyses were carried out with IBM SPSS Statistics 21.0. When we investigated if the promoter methylation status of *ABCB1*, *ABCC1* and/or *ABCG2* is associated with clinicopathological characteristics of the breast cancer patients (Table 2), methylation data was treated as categorical variable by dividing the levels into two categories, “methylation status < LOQ” and “methylation status ≥ LOQ”. If the methylation status was treated as continuous variable, methylation levels < LOQ (< 5%) were substituted with a default value, namely half the LOQ (2.5%), as proposed previously [58]. Chi-squared test was used to evaluate if the methylation status of individual CpGs in the promoter was associated with any of the clinicopathological parameters. One-way ANOVA (analysis of variance), followed by post-hoc Tukey's HSD (honest significant difference) test was applied to test for significant differences in the DNA methylation status between tumor, tumor-adjacent and tumor-distant tissues as well as tissues from the healthy control group. Pearson's correlation coefficient was used to assess the relationship between two continuous variables. In all cases, a *p value* < 0.05 (two-sided) was considered significant.

## SUPPLEMENTARY MATERIALS TABLES




