# Supplementary material for: Promoter methylation patterns of ABCB1, ABCC1 and ABCG2 in human cancer cell lines, multidrug-resistant cell models and tumor, tumor-adjacent and tumor-distant tissues from breast cancer patients
Source: Oncotarget. 2016 Sep 28;7(45):73347–69. doi: 10.18632/oncotarget.12332 (PMC5341984; doi:10.18632/oncotarget.12332)
Supplement: Supplementary file 1 [file oncotarget-07-73347-s001.pdf]

Promoter methylation patterns of *ABCB1, ABCC1* and *ABCG2* in human cancer cell lines, multidrug-resistant cell models and tumor, tumor-adjacent and tumor-distant tissues from breast cancer patients

Supplementary Materials

Supplementary Table S1: Details of cultured cell lines. See Supplementary_Table_S1

Supplementary Table S2: Primary antibodies used for Western Blotting

Primary antibody	Species	Clone	Dilution	Source
β-actin	Monoclonal mouse	AC-15	1:5000	Sigma Aldrich
ABCC1	Monoclonal rat	MRPr1	1:20	Alexis Biochemicals
ABCG2	Monoclonal mouse	BXP-21	1:500	Chemicon international
ABCB1	Mouse	C219	1:100	Calbiochem
