# Supplementary material for: Promoter methylation patterns of ABCB1, ABCC1 and ABCG2 in human cancer cell lines, multidrug-resistant cell models and tumor, tumor-adjacent and tumor-distant tissues from breast cancer patients
Source: Oncotarget. 2016 Sep 28;7(45):73347–69. doi: 10.18632/oncotarget.12332 (PMC5341984; doi:10.18632/oncotarget.12332)
Supplement: Supplementary file 2 [file oncotarget-07-73347-s002.docx]

Supplementary Table S1: Details of cultured cell lines

| **Cell line** | **Tissue origin** | **Disease** | **Growth medium** | **Source** |
| --- | --- | --- | --- | --- |
| A549 | Lung | NSCLC, adenocarcinoma | RPMI-1640 | ATCC |
| A2780 | Ovary | Ovarian carcinoma | RPMI-1640 | Sigma Aldrich |
| DMS114 | Lung | SCLC, carcinoma | RPMI-1640 | Dr. Sommergruber via Walter Berger |
| GLC-4 | Lung | SCLC, carcinoma | RPMI-1640 | E.G. deVries, Groningen, The Netherlands |
| GLC-4/adr | Adriamycin-selected, ABCC1-overexpressing subclone of GLC-4 |  | RPMI-1640 | E.G. deVries, Groningen, The Netherlands |
| HCC827 | Lung | NSCLC, adenocarcinoma | RPMI-1640 | ATCC |
| HCT116 | Colon | Colorectal carcinoma | McCoy´s | B. Vogestein, John Hopkins University, Baltimore; USA |
| HL-60 | Peripheral blood | Acute promyelocytic leukemia | RPMI-1640 | M. Center, Kansas State University, KS, USA |
| HL-60/adr | Adriamycin-selected, ABCC1-overexpressing subclone of HL60/adr |  | RPMI-1640 | M. Center, Kansas State University, KS, USA |
| HL-60/vinc | Vincristine-selected, ABCB1-overexpressing subclone of HL60/adr |  | RPMI-1640 | M. Center, Kansas State University, KS, USA |
| KB-3-1 | Cervix (HeLa contaminated) | Cervix carcinoma | RPMI-1640 | D. W. Shen, Bethesda, Maryland, USA |
| KBC-1 | Colchicine-selected, ABCB1-overexpressing subclone of KB-3-1 |  | RPMI-1640 | D. W. Shen, Bethesda, Maryland, USA |
| KB-1089 | KB-1089-selected, ABCC1- and ABCG2-overexpressing subclone of KB-3-1 |  | RPMI-1640 | in-house [[1](#_ENREF_1)] |
| MCF-7 | Mammary gland, breast; derived from metastatic site: pleural effusion | Adenocarcinoma | DMEM | ATCC |
| MDA-MB-231 | Mammary gland/breast; derived from metastatic site: pleural effusion | Adenocarcinoma | Leibovitz's L-15 | ATCC |
| MG-63 | Bone | Osteosarcoma | RPMI-1640 | ATCC |
| NCI-H520 | Lung | NSCLC, squamous cell carcinoma | RPMI-1640 | ATCC |
| NCI-H1703 | Lung | NSCLC, squamous cell carcinoma | RPMI-1640 | ATCC |
| PC-3 | Prostate; derived from metastatic site: bone | Adenocarcinoma | F-12K | ATCC |
| SW1573 | Lung | NSCLC, adenocarcinoma | DMEM | H. Broxterman, Department of Medical Oncology, Free University Hospital, Amsterdam, The Netherlands |
| SW1573/2R120 | Adriamycin-selected, ABCC1-overexpressing subclone of SW1573 |  | DMEM | H. Broxterman, Department of Medical Oncology, Free University Hospital, Amsterdam, The Netherlands |
| SW1573/2R160 | Adriamycin-selected, ABCB1- and ABCC1-overexpressing subclone of SW1573 |  | DMEM | H. Broxterman, Department of Medical Oncology, Free University Hospital, Amsterdam, The Netherlands |
| SW480 | Colon | Colorectal adenocarcinoma | MEME | ATCC |
| SW480/tria | Triapine-selected, ABCB1-overexpressing subclone of SW480 |  | MEME | in-house [[2](#_ENREF_2)] |
| U2-OS | Bone | Osteosarcoma | IMDM | ATCC |
| U266 | Peripheral blood; myeloma | Myeloma | RPMI-1640 | ATCC |
| ZR-75-1 | Mammary gland; breast/duct; derived from metastatic site: ascites | Ductal carcinoma | RPMI-1640 | ATCC |

ATCC: American Tissue Culture Collection, DMEM: Dulbecco´s Minimal Essential Medium, IMDM: Iscove's Modified Dulbecco's Medium, MEME: Minimal Essential Medium, NSCLC: non-small cell lung cancer; SCLC: small cell lung cancer;

1. Heffeter P, Pirker C, Kowol CR, Herrman G, Dornetshuber R, Miklos W, Jungwirth U, Koellensperger G, Keppler BK and Berger W. Impact of terminal dimethylation on the resistance profile of alpha-N-heterocyclic thiosemicarbazones. Biochemical Pharmacology. 2012; 83(12):1623-1633.

2. Miklos W, Pelivan K, Kowol CR, Pirker C, Dornetshuber-Fleiss R, Spitzwieser M, Englinger B, van Schoonhoven S, Cichna-Markl M, Koellensperger G, Keppler BK, Berger W and Heffeter P. Triapine-mediated ABCB1 induction via PKC induces widespread therapy unresponsiveness but is not underlying acquired triapine resistance. Cancer Letters. 2015; 361:112–120.
